# A dose- and schedule-finding study of darbepoetin alpha for the treatment of chronic anaemia of cancer

**DOI:** 10.1038/sj.bjc.6600994

**Published:** 2003-06-10

**Authors:** R E Smith, N S Tchekmedyian, D Chan, L A Meza, D W Northfelt, R Patel, M Austin, A B Colowick, G Rossi, J Glaspy

**Affiliations:** 1 South Carolina Oncology Associates, 1301 Taylor, Suite 1A, Columbia, SC 29201, USA; 2 Pacific Shores Medical Group, 1043 Elm Avenue, Suite 104, Long Beach, CA 90813, USA; 3 University of California Los Angeles, 514 N Prospect Ave 4th Fl, Redondo Beach, CA 90277, USA; 4 Southwest Oncology Associates Ltd, 443 Heymann Blvd, Suite A, Lafayette, LA 70503, USA; 5 Mayo Clinic, Hematology Oncology, 13400 East Shea Blvd, Scottsdale, AZ 85259, USA; 6 Comprehensive Blood and Cancer Center, 6501 Truxtun Ave, Bakersfield, CA 93309-0656, USA; 7 Amgen Inc., One Amgen Center Drive, Thousand Oaks, CA 91320, USA; 8 200 UCLA Medical Plaza, Suite 202, Los Angeles, CA 90095-6956, USA

**Keywords:** anaemia, chronic disease, darbepoetin alpha, erythropoietin

## Abstract

A multicentre study evaluated the efficacy and safety of darbepoetin alpha administered weekly (QW), every 3 weeks (Q3W), and every 4 weeks (Q4W) to anaemic patients with cancer not concurrently receiving chemotherapy or radiotherapy. The QW portion (*n*=102) was an open-label, sequential, dose-escalation design; cohorts received darbepoetin alpha QW by subcutaneous (s.c.) injection at 0.5, 1.0, 2.25, or 4.5 *μ*g kg^−1^ week^−1^ for 12 weeks. The 12-week placebo-controlled, double–blind Q3W (6.75 *μ*g kg^−1^) and Q4W (6.75 or 10.0 *μ*g kg^−1^) schedules (*n*=86), which enrolled different patients, took place after the QW schedule and were followed by a 12-week, open-label phase. Patients were evaluated for change in haemoglobin end points and red blood cell transfusions, serum darbepoetin alpha concentration, and safety. Selected domains of health-related quality of life (HRQOL) were measured. With QW dosing, at least 70% of each cohort had a haemoglobin increase from baseline of ⩾2 g dl^−1^ or a concentration ⩾12 g dl^−1^ (haematopoietic response). In the 4.5 *μ*g kg^−1^ QW cohort, all patients achieved a haematopoietic response (100%; 95% confidence interval (CI)=100, 100). In the Q3W and Q4W schedules, all cohorts had at least 60% of patients who achieved a haematopoietic response. Darbepoetin alpha effectively increases haemoglobin concentration when given QW, Q3W, or Q4W. Less-frequent administration may benefit patients with chronic anaemia of cancer and their caregivers alike.

Patients with cancer commonly develop anaemia, even if they are not receiving chemotherapy or radiotherapy. Anaemia in these patients may be the result of blood loss, haemolysis, bone marrow malfunction because of invasion with tumour, or other factors. Frequently, however, the cause is the anaemia of chronic disease (Cartwright, 1966; Lee, 1983). Patients with anaemia of chronic disease have a shortened red blood cell survival for which erythropoiesis fails to compensate (Miller *et al*, 1990; Koeller, 1998); experimental evidence indicates that inflammatory cytokines are responsible for the disruption of normal erythropoiesis (Means *et al*, 1995; Jelkmann, 1988). This occurs through several characteristic mechanisms. Iron utilisation appears defective, as indicated by typically low serum iron in the presence of adequate or elevated iron stores (Lee, 1983; Means and Kranz, 1992; Moliterno and Spivak, 1996). In addition, bone marrow erythroid progenitor cells show a blunted response to erythropoietin, with insufficient proliferation and a low circulating reticulocyte count (Means and Kranz, 1992; Moliterno and Spivak, 1996; Nowrousian, 1998). Finally, the production of erythropoietin itself in response to anaemia is inadequate. Compared to patients without cancer who have iron-deficiency anaemia, anaemic cancer patients have a smaller increase in erythropoietin for their level of anaemia (Ozguroglu *et al*, 2000).

Although anaemia of chronic disease is often mild to moderate in severity, this degree of anaemia and the associated symptoms can have considerably adverse effects on the lives of patients already in poor health. Fatigue, dyspnoea, and weakness have been shown to significantly affect the quality of daily life in patients with cancer (Cella, 1997a; Vogelzang *et al*, 1997; Curt, 2000). Studies in patients who have low haemoglobin levels for extended periods due to chronic renal insufficiency point to untreated anaemia as an independent risk factor for cardiovascular disease (Foley *et al*, 1996). Treatment with recombinant human erythropoietin (rHuEPO) earlier in the course of renal failure and correction of haemoglobin to higher levels are topics of current debate and investigation within the renal disease community, as some evidence suggests this approach may prevent the development or worsening of cardiac damage including left ventricular hypertrophy (NKF Anemia Work Group, 1997; Obrador *et al*, 1999; Hayashi *et al*, 2000; Macdougall, 2000).

An initial study in patients with cancer who were not receiving chemotherapy indicated that rHuEPO can correct anaemia in this population as well, with 32% of patients achieving a haematocrit increase of ⩾6% when rHuEPO was given at 100 U kg^−1^ three times weekly for 8 weeks (Abels *et al*, 1991). A significant reduction in red blood cell transfusions was not achieved at this dose and schedule; however, in a subsequent study using a higher dose of rHuEPO and longer duration of administration (150 U kg^−1^ three times weekly for up to 12 weeks) (Quirt *et al*, 2001), the percentage of nonchemotherapy patients requiring transfusions was significantly decreased by the second month of treatment. In the latter study, 48% of patients (39% of whom required a doubling of dose at week 5) met the definition for haemoglobin response (an increase in haemoglobin of at least 2 g dl^−1^ without transfusion).

Darbepoetin alpha is a protein that stimulates erythropoiesis in the same manner as rHuEPO, but has an increased number of sialic acid residues. The sialic acid-containing carbohydrate of erythropoietin determines its serum half-life (Egrie *et al*, 1993), and darbepoetin alpha has demonstrated a two- to three-fold longer serum half-life than rHuEPO in animal models and in patients with chronic renal failure (MacDougall *et al*, 1999; Egrie and Browne, 2001). In cancer patients undergoing chemotherapy, darbepoetin alpha therapy increased haemoglobin concentration when administered weekly (QW), or every 2 (Q2W), 3 (Q3W), or 4 (Q4W) weeks (Hedenus *et al*, 2002; Kotasek *et al*, 2002; Glaspy *et al*, 2002).

The study described here was the first to evaluate the dose, schedule, and pharmacokinetic profile of darbepoetin alpha in patients with anaemia of cancer, a condition for which dosing at weekly intervals or greater would be valuable. Darbepoetin alpha was given at one of four QW doses (from 0.5 to 4.5 *μ*g kg^−1^); at 6.75 *μ*g kg^−1^ Q3W; and at 6.75 or 10.0 *μ*g kg^−1^ Q4W.

## MATERIALS AND METHODS

### Study population

The institutional review boards for each of the 24 participating centres in the US approved the protocol and all patients provided written informed consent before any study-specific procedures were done. Men or women who were ⩾18 years of age with nonmyeloid malignancies were eligible for this study if they had anaemia (haemoglobin concentration ⩽11.0 g dl^−1^) due to either cancer or previous chemotherapy or radiotherapy (patients were required to have serum folate ⩾2.0 ng ml^−1^ and vitamin B_12_ ⩾200 pg ml^−1^, no haemolysis, and no gastrointestinal bleeding). Patients were not currently receiving chemotherapy or radiotherapy or planning to receive any for at least 16 weeks. A ⩾3-month life expectancy was required, as were an Eastern Cooperative Oncology Group (ECOG) performance status of 0–2, adequate renal function (serum creatinine concentration ⩽176.8 *μ*mol l^−1^) and liver function (serum bilirubin ⩽1.5 times the central laboratory upper limit of normal), and a platelet count of ⩾50 × 10^9^ l^−1^. Iron therapy and red blood cell transfusion policies were left to the discretion of the investigators, although transfusions were recommended at a haemoglobin level of ⩽8.0 g dl^−1^.

Patients were excluded from the study if they had both transferrin saturation <15% and ferritin <10 ng ml^−1^; had significant central nervous system, cardiac, or inflammatory diseases; or had known primary haematologic disorders that could cause anaemia. Patients were also excluded if they had received chemotherapy or rHuEPO within 8 weeks before enrolment, any red blood cell transfusion within 16 days of enrolment, or >2 red blood cell transfusions within 4 weeks of enrolment.

### Study design

This was a multicentre, dose- and schedule-finding study in patients with nonmyeloid tumours who were not currently receiving chemotherapy. Three schedules were evaluated; darbepoetin alpha administered QW, Q3W, and Q4W. Different patients were enrolled in each schedule. In the QW schedule, patients had 12 weeks of open-label therapy with darbepoetin alpha given subcutaneously (s.c.) once weekly at sequentially escalated doses of 0.5, 1.0, 2.25, or 4.5 *μ*g kg^−1^. The Q3W and Q4W schedules took place after the completion of the QW schedule, and were double-blind and placebo-controlled. Patients were randomised in a 3 : 1 ratio to receive darbepoetin alpha (6.75 *μ*g kg^−1^ Q3W, 6.75 *μ*g kg^−1^ Q4W or 10.0 *μ*g kg^−1^ Q4W) or placebo (Q3W or Q4W) S.C. for the first 12 weeks (blinded treatment phase), and could then participate in an optional, 12-week, open-label darbepoetin alpha treatment phase with a 4-week observation period after the last dose. Placebo patients were assigned the dose of darbepoetin alpha for which they served as the control during the blinded phase.

No dose increase for inadequate response was allowed in this study. Study drug was to be withheld if a patient's haemoglobin value increased to >15.0 g dl^−1^ for men or >14.0 g dl^−1^ for women. Once the haemoglobin value was ⩽13.0 g dl^−1^, study drug could be reinstated at a lower dose. Initially in the QW schedule, an increase in haemoglobin concentration of ⩾2.0 g dl^−1^ in the absence of a red blood cell transfusion during the previous 28 days was used as a criterion for dose reductions. This criterion was amended to add the requirement that the haemoglobin concentration also be >13.0 g dl^−1^. If the dose was reduced, the patient remained at that dose until the end of the 12-week treatment period.

Predose and 48-h postdose serum samples for determination of darbepoetin alpha concentration were collected in a subset of patients at weeks 1, 4, and 12 (QW); 1, 7, and 13 (Q3W); and 1, 5, and 13 (Q4W).

### Study drug

Darbepoetin alpha (Aranesp®, Amgen Inc., Thousand Oaks, CA, USA) was supplied in vials as a clear, colourless, sterile protein containing 100, 300, 500, 1000, or 1500 *μ*g darbepoetin alpha per ml. Placebo was supplied in identical vials.

### Study endpoints

All three schedules evaluated the potential efficacy of darbepoetin alpha. Efficacy was assessed as the incidence of patients achieving haematopoietic response, defined as an increase in haemoglobin of ⩾2.0 g dl^−1^ from baseline during the 12-week treatment period, or a haemoglobin concentration ⩾12.0 g dl^−1^, in the absence of any red blood cell transfusions during the previous 28 days. This end point is a commonly used measurement of response in other reports in the literature (Demetri *et al*, 1998; Gabrilove *et al*, 2001). Other haemoglobin end points were the time to haematopoietic response, the incidence of patients achieving a haemoglobin response (an increase in haemoglobin concentration of ⩾2.0 g dl^−1^ from baseline during the 12-week treatment period, in the absence of any red blood cell transfusions during the previous 28 days), and the change in haemoglobin from baseline during treatment.

The incidence of red blood cell transfusions was assessed using a subset of the analysis set. In other studies evaluating red blood cell transfusion requirements (Abels, 1992; Dammacco *et al*, 2001; Littlewood *et al*, 2001), treatment effects were not observed until after 4 weeks of treatment. Therefore, the incidence of transfusions was analysed from week 5 to the end of the treatment period.

Serum darbepoetin alpha concentration was determined in a subset of patients.

Safety was assessed in each schedule by summarising the incidence of adverse events by dose and treatment group, and by evaluating the formation of antibodies resulting from darbepoetin alpha administration. A radioimmunoprecipitation screening assay was used to detect seroreactivity to darbepoetin alpha, and a cell-based bioassay to detect neutralising or inhibiting effects on the activity of darbepoetin alpha.

The primary health-related quality-of-life (HRQOL) scale in this study was the FACT-Fatigue subscale score, which has been validated with oncology patients (Cella, 1997a). Patients completed the HRQOL questionnaire (Cella, 1997b), which included the FACT-An (anaemia/fatigue) scales (FACT-General scales and the FACT-Fatigue subscale), every 4 weeks before any other study procedures.

### Statistical analysis

Except for red blood cell transfusions, statistical analyses were carried out on the set of patients who received at least one dose of study drug. Patients who had more than one transfusion were counted only once in calculating the incidence of transfusions.

Baseline demographic and clinical characteristics were summarised by using the mean and standard deviation (s.d.) for continuous measures and the number and percent for categorical measures. The number and proportion of patients in each dose group were calculated for haematopoietic response, haemoglobin response, and incidence of transfusions from week 5 to the end of the treatment period (week 12 in the QW schedule, week 12 of the blinded phase in the Q3W/Q4W schedules). The proportion was estimated by taking 1 minus the Kaplan-Meier (K-M) estimate of the survivor function at the time of the last observed endpoint (the K-M estimate). Approximate 95% confidence intervals (CIs) for the K-M estimate were calculated using Greenwood's (Kalbfleisch and Prentice, 1980) estimate of the variance and assuming a normal distribution of the survivor function.

Time to haematopoietic response was calculated as the time from study day 1 to the first occurrence of the relevant haemoglobin end point.

The change in haemoglobin concentration (mean and standard error (s.e.)) was assessed by (1) subtracting the baseline haemoglobin value from the end-of-treatment-phase value for each patient (all patients had an observed or imputed value for this analysis), (2) using the set of patients who completed at least 12 weeks of treatment, and (3) for the Q3W and Q4W schedules, as the end of the open-label phase value minus the beginning of the open-label phase value for each patient. If a patient had a transfusion within 28 days before the last treatment phase haemoglobin value, the last pretransfusion haemoglobin value was substituted to discount the effect of the transfusion on haemoglobin concentration.

The number (%) of patients experiencing adverse events and the number (%) of patients with antibodies resulting from darbepoetin alpha administration were tabulated. The pharmacokinetic analysis was performed using noncompartmental methods.

## RESULTS

### Patient demographics and disposition

**Figure 1:**
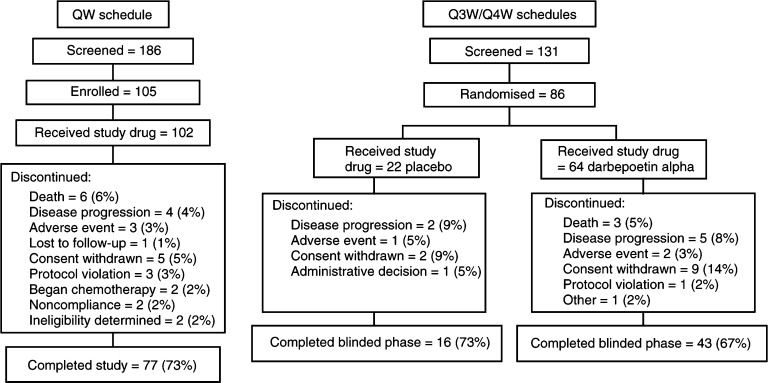
Disposition of patients.

A total of 102 patients in the QW schedule and 86 patients in the Q3W/Q4W schedules (64 darbepoetin alpha and 22 placebo) received at least one dose of study drug (Figure 1

). Only six patients were enrolled into the 0.5 *μ*g kg^−1^ cohort of the QW schedule (the total number on study at the time the Data Monitoring Committee recommended escalation to the next dose cohort as the specified safety criteria were met).

Baseline demographic and clinical characteristics of patients were generally well balanced between the cohorts including the placebo cohort (Table 1

**Table 1:** Baseline demographic and clinical characteristics: (a) QW schedule, (b) Q3W and Q4W schedules

	**(A) Darbepoetin alpha dose (*μ*g kg^−1^ QW)**				**(B) Treatment group – placebo or darbepoetin alpha**			
	**0.5 (*N*=6)**	**1.0 (*N*=33)**	**2.25 (*N*=33)**	**4.5 (*N*=30)**	**Placebo (*N*=22)**	**6.75 Q3W (*N*=21)**	**6.75 Q4W (*N*=21)**	**10.0 Q4W (*N*=22)**
Age (years)								
Mean (s.d.)	53.2 (8.6)	69.7 (13.5)	70.2 (11.0)	74.0 (9.1)	68.8 (17.6)	64.0 (16.6)	70.5 (11.2)	68.8 (9.1)
Sex								
(*n*/%) women	5 (83)	14 (42)	17 (52)	12 (40)	13 (59)	18 (86)	13 (62)	11 (50)

Disease (*n*/%)								
Genitourinary	1 (17)	12 (36)	9 (27)	12 (40)	5 (23)	2 (10)	2 (10)	3 (14)
Breast	4 (67)	9 (27)	10 (30)	7 (23)	1 (5)	8 (38)	6 (29)	4 (18)
Lymphoid malignancy^a^	0 (0)	6 (18)	8 (24)	3 (10)	4 (18)	5 (24)	6 (29)	1 (5)
Gastrointestinal	0 (0)	1 (3)	2 (6)	4 (13)	4 (18)	2 (10)	2 (10)	4 (18)
Lung	0 (0)	1 (3)	2 (6)	2 (7)	2 (9)	2 (10)	3 (14)	5 (23)
Gynaecologic	0 (0)	1 (3)	0 (0)	1 (3)	2 (9)	1 (5)	1 (5)	4 (18)
Other	1 (17)	3 (9)	2 (6)	1 (3)	4 (18)	1 (5)	1 (5)	1 (5)

Disease stage (*n*/%)^b^								
Stages I–II	0 (0)	11 (33)	4 (12)	7 (23)	6 (27)	4 (19)	6 (29)	5 (23)
Stages III–IV	6 (100)	21 (64)	27 (82)	21 (70)	15 (68)	15 (71)	12 (57)	15 (68)
Unknown	0 (0)	1 (3)	2 (6)	2 (7)	1 (5)	2 (10)	3 (14)	2 (9)

Pretreatment chemotherapy or radiotherapy (*n*/%)	5 (83)	27 (82)	28 (85)	23 (77)	18 (82)	17 (81)	17 (81)	21 (95)

Haemoglobin (g dl^−1^)								
Mean (s.d.)	9.60 (1.53)	9.80 (0.91)	9.62 (1.12)	10.05 (0.94)	9.84 (0.89)	9.84 (1.38)	10.20 (0.88)	10.10 (0.80)

Endogenous EPO (mU ml^−1^)								
*N*	6	33	33	28	21	20	21	21
Median	42.93	32.03	30.94	23.46	26.42	46.22	29.32	28.99
Q1, Q3	24.08, 159.41	21.25, 54.06	18.80, 99.55	16.07, 34.60	16.31, 38.43	20.61, 61.17	17.66, 47.78	16.41, 45.98

Ferritin (*μ*g l^−1^)								
*N*	6	33	33	29	22	21	21	22
Median	1164.0	472.0	206.0	149.0	133.5	92.0	113.0	169.5
Q1, Q3	151.0, 2010.0	173.0, 942.0	97.0, 416.0	74.0, 402.0	36.0, 605.0	41.0, 292.0	50.0, 243.0	46.0, 465.0

a Includes myelomas, chronic lymphocytic leukaemia, and non-Hodgkin's lymphoma.

b Disease stage as defined by the investigator. QW=once weekly; Q3W=once every 3 weeks; Q4W=once every 4 weeks; *N*=number in cohort; s.d.=standard deviation; *n*=number in subset; EPO=erythropoietin; Q=quartile.

). The most frequent diagnosis was breast cancer, but a large proportion of patients with other malignancies (e.g., lung, gastrointestinal, gynaecologic, genitourinary) were also represented. The 6.75 *μ*g kg^−1^ Q3W cohort had a greater proportion of women and a higher median endogenous erythropoietin concentration at baseline compared with the other cohorts. A total of 156 (83%) patients had received prior chemotherapy or radiotherapy; of these, 112 (72%) had undergone their last chemotherapy or radiotherapy at least 6 months previously. All patients had undergone their last chemotherapy at least 8 weeks previously and were therefore eligible for the study. At least 80% of patients in each treatment group had an ECOG status of <2.

The disposition of patients enrolled into the trial is given in Figure 1. A total of 77 patients (73%) completed the QW schedule, while 43 (67%) patients receiving darbepoetin alpha and 16 (73%) patients receiving placebo completed the blinded portion of the Q3W/Q4W schedules. As expected in this population, death, disease progression, and/or intervention with chemotherapy or radiotherapy were frequent reasons for withdrawal from study. Fifty-nine patients enrolled into the open-label extension of the Q3W/Q4W schedules and received darbepoetin alpha. Six (6%) patients died during the QW schedule of the study, and five (5%) died during the Q3W/Q4W schedules (three during the blinded phase and two during the open-label phase). None of these deaths were considered related to study drug treatment.

### Study end points

#### Efficacy

*Incidence of patients achieving haematopoietic response*: With weekly dosing, the proportion of patients achieving haematopoietic response ranged from 70% (95% CI=53, 88) at the 2.25 *μ*g kg^−1^ dose to 100% (95% CI=100, 100) at the 4.5 *μ*g kg^−1^ dose (Table 2

**Table 2:** Haemoglobin end points and red blood cell transfusions

	**Treatment group – placebo or darbepoetin alpha (*μ*g kg^−1^)**							
	**0.5 QW**	**1.0 QW**	**2.25 QW**	**4.5 QW**	**Placebo**	**6.75 Q3W**	**6.75 Q4W**	**10.0 Q4W**
	**(*N*=6)**	**(*N*=33)**	**(*N*=33)**	**(*N*=30)**	**(*N*=22)**	**(*N*=21)**	**(*N*=21)**	**(*N*=22)**
Haematopoietic response								
Proportion^a^	80	72	70	100	10	60	61	70
(95% CI)	(45, 100)	(55, 89)	(53, 88)	(100, 100)	(0, 24)	(36, 83)	(39, 84)	(50, 91)
Median time to haematopoietic response (days)	50	50	36	27	NE	57	36	40
Haemoglobin response								
Proportion^a^	80	69	67	92	5	58	49	60
(95% CI)	(45, 100)	(51, 87)	(49, 85)	(80, 100)	(0, 15)	(34, 82)	(26, 73)	(39, 82)
Mean change in haemoglobin concentration (g dl^−1^) (imputed analysis)^b^								
Mean	1.45	1.66	2.07	2.91	0.00	1.18	1.22	1.70
95% CI	(−0.05,2.95)	(0.90,2.42)	(1.34,2.80)	(2.20, 3.62)	(−0.39,0.39)	(0.33,2.04)	(0.53,1.92)	(0.89,2.50)
Mean change in haemoglobin concentration (g dl^−1^) (alternate analysis)^b^								
*n*	4	20	22	18	16	15	15	13
Mean	1.98	2.01	2.69	3.16	0.05	1.71	1.38	2.05
95% CI	(−0.8,4.75)	(0.85,3.18)	(1.83,3.55)	(2.49,3.84)	(−0.40,0.50)	(0.73,2.69)	(0.48,2.28)	(1.5,3.04)
Patients with RBC transfusion from week 5 to EOTP (subset analysis)								
*n*	5	32	30	29	20	20	20	21
Proportion^a^	20	36	14	7	21	16	6	10
(95% CI)	(0, 55)	(10, 62)	(1, 26)	(0, 17)	(3, 39)	(0, 33)	(0, 16)	(0, 23)

a Proportion calculated using K-M estimate.

b Change from baseline calculated using last available haemoglobin value not within 28 days of a transfusion (imputed analysis), and for patients with haemoglobin value at week 13 not within 28 days of a transfusion (alternate analysis). QW=once weekly; Q3W=once every 3 weeks; Q4W=once every 4 weeks; *N*=number in cohort; CI=confidence interval; RBC=red blood cell; EOTP=end of treatment period; *n*=number in subset; NE=not estimable (median not reached).

, Figure 2

**Figure 2:**
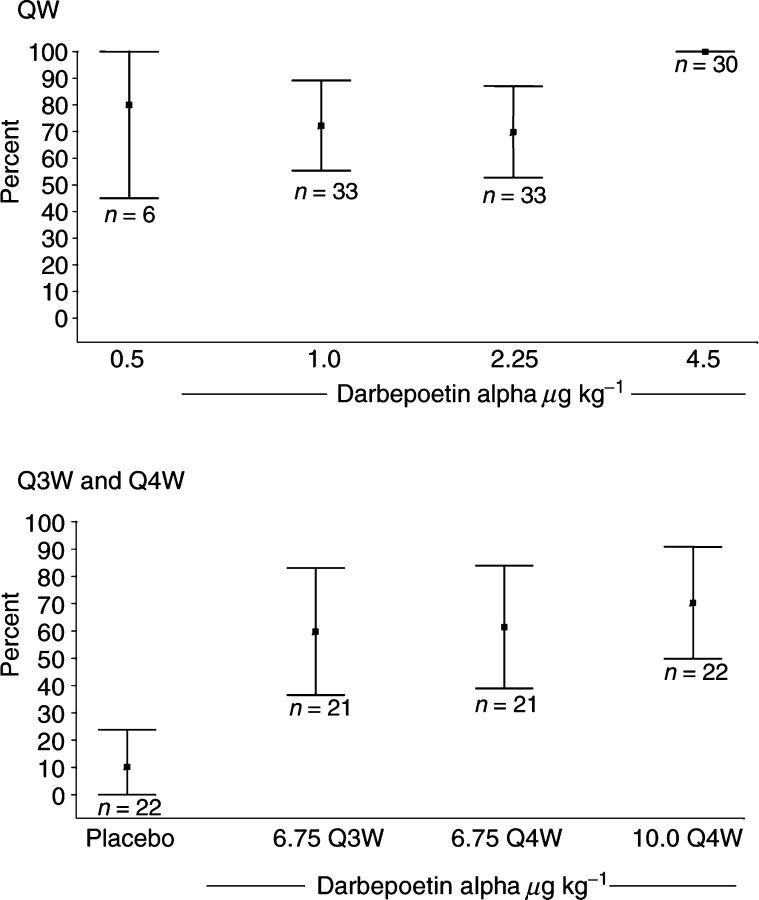
Proportion of patients with haematopoietic response.

). In the Q3W/Q4W schedules, the haematopoietic response ranged from 60% (95% CI=36, 83) to 70% (95% CI=50, 91) in the darbepoetin alpha cohorts and was 10% (95% CI=0, 24) in the placebo cohort (Table 2, Figure 2).

*Time to haematopoietic response*: The QW data suggested a dose–response relationship for time to haematopoietic response, although none was apparent in the Q3W/Q4W schedules. The median time to haematopoietic response was not reached in the placebo group, and ranged from 27 to 57 days across the other cohorts, with the 4.5 *μ*g kg^−1^ QW cohort responding the fastest (Table 2).

*Incidence of patients achieving a haemoglobin response*: In the QW schedule, at least 67% of patients in each cohort achieved a haemoglobin response, with the highest proportion of responders (92%; 95% CI=80, 100) at the 4.5 *μ*g kg^−1^ dose (Table 2). In the Q3W and Q4W schedules, the 6.75 *μ*g kg^−1^ Q3W and 10.0 *μ*g kg^−1^ Q4W groups had a similar proportion of responders (58% (95% CI=34, 82) and 60% (95% CI=39, 82), respectively), with 49% (95% CI=26, 73) of patients in the 6.75 *μ*g kg^−1^ Q4W group responding. One patient who received placebo (5%; 95% CI=0, 15) had a haemoglobin response.

*Change in haemoglobin*: In the QW schedule, a dose-response relationship was suggested by the mean change in haemoglobin concentration from baseline to the end of the treatment period, with the greatest increase (2.91 g dl^−1^; 95% CI=2.20, 3.62) at the 4.5 *μ*g kg^−1^ dose (Table 2). In the Q3W and Q4W schedules, each darbepoetin alpha cohort had a mean increase in haemoglobin concentration of at least 1.0 g dl^−1^ during the 12 weeks of blinded treatment, while mean haemoglobin concentration in the placebo group was unchanged. When analysing only those patients who completed at least 12 weeks of treatment (i.e., had a haemoglobin value at week 13 with no transfusion during the preceding 28 days), the mean increases range from 1.98 g dl^−1^ (95% CI=−0.8, 4.75) to 3.16 g dl^−1^ (95% CI=2.49, 3.84) with QW dosing, and 1.38 g dl^−1^ (95% CI=0.48, 2.28) to 2.05 g dl^−1^ (95% CI=1.5, 3.04) with Q3W/Q4W dosing.

**Figure 3:**
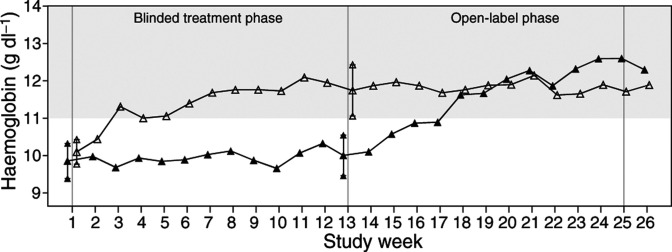
Mean change in haemoglobin over double-blind and open-label phases of Q3W and Q4W schedules.

Patients who received darbepoetin alpha in the blinded phase and then entered the open-label phase continued to receive darbepoetin alpha at the same dose/schedule for 12 more weeks while maintaining their haemoglobin concentration (Figure 3

). Patients who received placebo during the blinded phase of the study and then crossed over to active treatment during the open-label phase had an increase in haemoglobin concentration over their 12 weeks of open-label darbepoetin alpha treatment, with mean gains during these 12 weeks of 2.42 g dl^−1^ (95% CI=−0.12, 4.96) at 6.75 *μ*g kg^−1^ Q3W, *n*=5; 1.77 g dl^−1^ (95% CI=−0.13, 3.67) at 6.75 *μ*g kg^−1^ Q4W, *n*=7; and 1.98 g dl^−1^ (95% CI=0.51, 3.46 at 10.0 *μ*g kg^−1^ Q4W, *n*=6.

*Red blood cell transfusions*: While the eligibility criteria excluded patients who were transfusion dependent, the incidence of transfusions was assessed over the course of the study. A trend towards fewer transfusions at the higher darbepoetin alpha dose levels was apparent in the QW schedule (Table 2). In the Q3W/Q4W schedules, 21% (95% CI=3, 39) of patients receiving placebo required a red blood cell transfusion, while the proportions for the darbepoetin alpha cohorts ranged from 6% (95% CI=0, 16) to 16% (95% CI=0, 33).

#### HRQOL

The FACT-Fatigue subscale had a mean baseline score of 26.9 (out of a possible maximum of 52 points) with a mean change of 8.5 points (95% CI=5.9, 11.1) for patients who had a ⩾2 g dl^−1^ increase in haemoglobin compared to a 0.6-point decline (95% CI: −6.0, 4.8) in patients with no increase in haemoglobin concen-tration. Results from the composite scales all showed similar trends with significant increases in scale score in patients with a ⩾2 g dl^−1^ increase in haemoglobin from baseline. The relationship with increasing haemoglobin concentration was observed for the FACT-Fatigue subscale (Jonckheere–Terpstra test *P*-value <0.01) as well as other FACT-General subscales except for the FACT-Social/Family well-being and FACT-Emotional subscales (Table 3

**Table 3:** Change in FACT scales with change in haemoglobin concentration (QW, Q2W, and Q3W schedules combined)

	**Haemoglobin change category (baseline to end of treatment period)**			
	**Baseline value**	**0 g dl^−1^**	**0 g/dL–2 g dl^−1^**	**⩾ 2g dl^−1^**
**FACT scales**	***n*, Mean (95% CI)**	**Mean (95% CI)**	**Mean (95% CI)**	**Mean (95% CI)**
*n*	183^a^	22	76^b^	85^c^
FACT Fatigue subscale	26.9 (25.0, 28.8)	−0.6 (−6.0, 4.8)	1.7 (−1.1, 4.5)	8.5 (5.9, 11.1)
FACT Anaemia symptoms subscale	17.8 (17.0, 18.5)	−1.4 (−4.3, 1.5)	0.8 (0.0, 1.6)	2.5 (1.5, 3.5)

FACT Anaemia subscale score^d^	44.6 (42.2, 47.1)	−2.0 (−9.7, 5.7)	2.5 (−0.8, 5.9)	11.0 (7.8, 14.2)
				
FACT-G Physical well-being subscale	19.3 (18.4, 20.1)	−1.2 (−3.5, 1.1)	0.4 (−0.9, 1.6)	2.8 (1.6, 4.0)
FACT-G Social/Family well-being subscale	21.6 (20.7, 22.5)	−0.4 (−2.9, 2.0)	−0.2 (−1.6, 1.1)	−0.4 (−1.4, 0.6)
FACT-G Emotional well-being subscale	17.5 (16.8, 18.3)	−0.2 (−2.3, 2.0)	0.8 (−0.1, 1.7)	1.5 (0.7, 2.3)
FACT-G Functional well-being subscale	14.6 (13.6, 15.6)	−2.1 (−4.6, 0.4)	0.0 (−1.3, 1.3)	3.6 (2.4, 4.9)

FACT-G Overall scale^e^	73.0 (70.4, 75.6)	−3.9 (−9.7, 2.0)	1.2 (−1.9, 4.3)	7.6 (4.6, 10.6)

FACT Anaemia total scale^f^	117.5 (112.7, 122.2)	−5.9 (−18.6, 6.9)	4.1 (−1.6, 9.8)	18.7 (12.9, 24.6)

a *n*=182 for the FACT-G Functional scale, 179 for the Social/Family scale, and 178 for the Overall and Anaemia Total scales.

b *n*=73 for the FACT-G Social/Family, Overall, and Anaemia Total scales.

c *n*=84 for the FACT-G Social/Family and Functional scales and 83 for the Overall and Anaemia total scales.

d Anaemia subscale score=Fatigue+Anaemia symptoms.

e FACT-G Overall score=Physical well-being+Social/family well-being+Emotional well-being+Functional well-being.

f FACT Anaemia total score=FACT-G+Anaemia subscale score.

). As the FACT-Social/Family well-being subscale questions focus on social support and family communication, this subscale was not hypothesised to be correlated with either the symptoms of anaemia or outcomes resulting from its treatment.

#### Pharmacokinetics

As assessed in seven patients at the 6.75 *μ*g kg^−1^ dose, serum concentrations of darbepoetin alpha remained at effective levels for 2–3 weeks and returned to baseline values within approximately 3 weeks. Key pharmacokinetic parameters determined at this dose were mean area under the curve (from time 0 to infinity) 3760 ng h ml^−1^, mean half-life 54.5 h; and time to maximum concentration 47.9 h.

#### Safety

**Figure 4:**
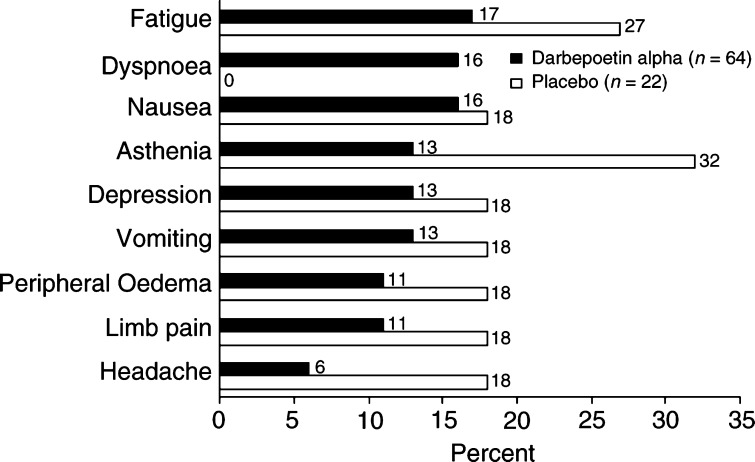
Adverse events that occurred with ⩾15% incidence in either treatment group in the Q3W and Q4W schedules.

Safety was assessed by summarising the incidence of adverse events by dose and treatment group. In general, the safety profile of darbepoetin alpha was similar for all three schedules of administration. The most frequently reported adverse events in the QW schedule were fatigue (24%), arthralgia (19%), and asthenia (19%). In the Q3W/Q4W schedules, the most frequent events were fatigue (17% darbepoetin alpha; 27% placebo), dyspnoea (16% darbepoetin alpha; 0% placebo), and nausea (16% darbepoetin alpha; 18% placebo) (Figure 4

).

No relation between dose and adverse events was noted. Twenty-seven patients receiving darbepoetin alpha in the QW schedule and eight in the blinded phase of the Q3W/Q4W schedules reached the maximum haemoglobin concentration. Of these, 26 patients had study drug withheld according to the protocol; in general, haemoglobin concentrations decreased or stabilised in these patients.

Twenty-four patients reached a haemoglobin concentration >13 g dl^−1^ accompanied by a ⩾2.0 g dl^−1^ increase without previous transfusion (12 patients in the QW schedule; seven darbepoetin alpha patients and no placebo patients in the blinded portion of the Q3W/Q4W schedules; five patients in the open-label portion). Too few patients met this criterion to adequately evaluate potential differences in the incidence and types of adverse events.

Four patients (3.9%) had thrombotic events during the QW schedule (three with lower extremity deep vein thrombosis (DVT) and one with a pulmonary embolism and a DVT). In the Q3W/Q4W schedules, one placebo patient developed a bilateral lower extremity DVT, and one patient in the open-label 10.0 *μ*g kg^−1^ Q4W group developed a left lower extremity DVT and bilateral pulmonary emboli. None of these events were associated with a rapid increase in haemoglobin (as defined in the previous paragraph) or a protocol-specified maximum haemoglobin level (>14.0 g dl^−1^ for women, >15.0 g dl^−1^ for men).

No evidence of neutralising antibodies to darbepoetin alpha was detected.

## DISCUSSION

This was the first dose-finding study of an erythropoietic agent in patients with anaemia of cancer not concurrently being treated with chemotherapy. Eighty-three percent of the patients had received prior chemotherapy or radiotherapy; of these, 72% had received their last chemotherapy at least 25 weeks previously, and none more recently than 8 weeks previously, indicating that this was truly a population with anaemia of chronic disease rather than one experiencing the residual effects of recent chemotherapy. As would be expected in anaemia of chronic disease, laboratory indices indicated suppression of endogenous EPO and evidence of impaired iron utilisation. Although patients in the QW and Q3W/Q4W schedules were not enrolled contemporaneously, they can legitimately be compared because the same population of patients was studied and the study designs were similar. Few conclusions can be drawn about the 0.5 *μ*g kg^−1^ cohort because of its small sample size (*n*=6), and this dose is disregarded for the remainder of the discussion – although it is interesting that 80% of patients even at this low dose achieved a haemoglobin response.

Adverse events observed in this study were consistent with those expected for an elderly population of patients with cancer and chronic anaemia and were similar for all doses and schedules. The 16% incidence of dyspnoea in the Q3W/Q4W schedules is similar to that seen in the QW schedule (13%); the discrepancy with the placebo group may be an artefact of the small sample sizes, as dyspnoea would be expected to be relatively common in anaemic patients.

A robust response in haemoglobin levels was seen at all doses/schedules, whether QW, Q3W, or Q4W, with at least 60% of all cohorts achieving an increase of ⩾2 g dl^−1^ and/or a concentration ⩾12 g dl^−1^ in the absence of transfusions. Using the standard measures of efficacy, the QW dose cohorts (1.0–4.5 *μ*g kg^−1^ darbepoetin alpha QW) achieved response rates that were generally higher than those previously reported with rHuEPO. Haemoglobin response rates ranged from approximately 70 to 90% of patients, compared with 48% (39% of whom required a doubling of dose at week 5) in the recently reported study by Quirt *et al* (2001). A trend towards decreasing time to response with increasing dose was also observed with the QW schedule of administration for darbepoetin alpha. Mean haemoglobin concentration increased by 1.45 g dl^−1^ (95% CI=−0.05, 2.95) to 2.91 g dl^−1^ (95% CI=2.20, 3.62) across the various cohorts. For those patients who completed 12 weeks of treatment, the mean increase was generally 0.2–0.5 g dl^−1^ higher than the values obtained using the analysis that includes the last observed value for patients who withdrew from the study early.

Darbepoetin alpha was also shown to be effective when administered every 3 or 4 weeks compared with placebo. Haemoglobin response rates of approximately 50% or greater were observed despite the infrequent schedule, with haematopoietic response rates of between 60 and 70% with Q3W or Q4W administration. An evaluation of the efficiency of darbepoetin alpha as the schedule of administration decreases is possible in this study. At dose/schedules that administered an approximately equivalent cumulative dose of darbepoetin alpha, the impact on haemoglobin variables was broadly similar, for example, haematopoietic response was achieved by 70% (95% CI=53, 88) of patients at 2.25 *μ*g kg^−1^ QW; by 60% (95% CI=36, 83) at 6.75 *μ*g kg^−1^ Q3W; and by 70% (95% CI=50, 91) at 10 *μ*g kg^−1^ Q4W. Although the study was not designed to test individual doses against placebo, based on the (family-wise) 95% CI, a higher proportion of patients receiving darbepoetin alpha 6.75 *μ*g kg^−1^ Q4W (the lowest dose in the placebo-controlled portion of the study) met the criteria for haemoglobin and haematopoietic response than placebo patients, suggesting that this may be an effective monthly dose.

The mean haemoglobin increases for the placebo cohorts crossing over to darbepoetin alpha during the second 12 weeks were somewhat greater than those for the darbepoetin alpha groups during the first 12 weeks; this is probably the result of selection bias as the more debilitated patients withdrew from the study. That placebo patients had no increase in haemoglobin during the first 12 weeks provides additional evidence that this population was genuinely anaemic because of the anaemia of chronic disease and not just recovering from prior chemotherapy.

This study was not designed to conclusively evaluate the effect on transfusions, as it had a relatively small sample size to evaluate infrequently occurring events. The eligibility criteria minimised the entry of patients with any significant transfusion requirement, probably reducing the number of transfusions over the course of the 12-week study period as well, and consequently minimising differences between the placebo and treatment groups. The transfusion rates in the darbepoetin alpha groups are generally similar to those reported in the literature for patients treated with rHuEPO (Glaspy *et al*, 1997; Demetri *et al*, 1998; Littlewood *et al*, 2001; Quirt *et al*, 2001).

Similarly, because of the exploratory nature of this study, conclusive evidence regarding HRQOL cannot be provided. However, the relation between FACT-F scores, particularly the reduction in fatigue, and haemoglobin concentration seen here is consistent with other reports that have found improved alleviation of patient-reported symptoms of anaemia with increasing haemoglobin levels (Glaspy *et al*, 1997; Gabrilove *et al*, 2001). Confirmatory studies are needed to investigate whether the observed increases in haemoglobin translate into reductions in transfusion requirements and/or improvements in HRQOL.

Frequent dosing with rHuEPO may be particularly inconvenient for anaemic cancer patients who are not receiving chemotherapy, as it may create the need for clinic visits that would otherwise not be necessary, and introduces a burdensome regimen for those living with a potentially long-term condition. A direct comparison of rHuEPO and darbepoetin alpha would be of interest to better understand the relative advantages of less-frequent dosing and the comparative efficacy of the two agents. The findings from this phase 2 study have shown that darbepoetin alpha is effective compared with placebo using administration schedules as infrequent as every 4 weeks. Weekly administration of darbepoetin alpha resulted in up to 100% of patients achieving a response, and observed rates of response using Q3W and Q4W schedules were at least comparable to those seen with three-times-weekly rHuEPO in this population (Abels *et al*, 1991; Quirt *et al*, 2001). Based on the evidence from this study, even the lowest dose of darbepoetin alpha (6.75 *μ*g kg^−1^) administered Q4W demonstrated superior haematologic responses (e.g., change in haemoglobin, haemoglobin response) compared with placebo. In summary, darbepoetin alpha appears to be a potent agent for increasing haemoglobin concentration in anaemic patients with cancer not receiving chemotherapy, allowing almost all patients to achieve desired concentrations; less frequent administration provides significant benefits to patients, their caregivers, and the oncology office practice.

## Appendix 1.

Additional members (investigators) of the Aranesp 990111 Study Group

Mark J Adler (Oncology Medical Center of North County, Vista, CA, USA); Heather J Allen (Southwest Cancer Clinic - East Office, Las Vegas, NV, USA); John Barstis (UCLA/Antelope Valley Cancer Center, Lancaster, CA, USA); Barry Berman (Cancer Center of Florida, FL, USA); Douglas W Blayney (The Robert & Beverly Lewis Family Cancer Care Center, Pomona, CA, USA); Stanley A Brosman (Pacific Clinical Research, Santa Monica, CA, USA); Robert P Brouillard (La Jolla, CA, USA); Ronald Bukowski (Cleveland Clinic Foundation, Cleveland, OH, USA); Patrick J Cannon (Encino, CA, USA); Joseph F Chang (Bakersfield, CA, USA); Harry Griffith (Western Washington Oncology, Shelton, WA, USA); Julie Hersch (Santa Cruz, CA, USA); Ishmael Jaiyesimi (Beaumont Cancer Center, Royal Oak, MI, USA); Glen Justice (Pacific Coast Hematology Oncology Medical Group, Fountain Valley, CA, USA); Jay P Klarnet (Hematology Oncology Northwest, Tacoma, WA, USA); Richard Lloyd (Virginia K. Crosson Cancer Center, Fullerton, CA, USA); Rosemary E McIntyre (Ventura County Hematology Oncology Specialists, Oxnard, CA, USA); Harry Menco (Thousand Oaks, CA, USA); Myron Murdock (Drs Wemer, Murdock, Francis, PA, Urology Associates, Greenbelt, MD, USA); Christopher Perkins (California Oncology of the Central Valley, Fresno, CA, USA); James E Pero (Thousand Oaks, CA, USA); James L Pot (Santa Cruz, CA, USA); Mark Rarick (Hematology/Oncology, Kaiser Permanente, Portland, OR, USA); Donald Richards (Tyler Cancer Center, Tyler, TX, USA); Alan Saven (Scripps Clinic, Division of Hematology Oncology, La Jolla, CA, USA); Roger Shiffman (Monterey Bay Oncology, Monterey, CA, USA); and Thomas Woliver (Santa Barbara Hematology Oncology Medical Group, Inc, CA, USA).
